# Early immune responses to intradermal lipopolysaccharide in healthy volunteers: prednisolone’s impact on TLR4-mediated inflammation

**DOI:** 10.3389/fimmu.2026.1724791

**Published:** 2026-05-20

**Authors:** Thomas P. Buters, Digna T. de Bruin, Pieter W. Hameeteman, Wouter ten Voorde, Hendrika W. Grievink, Michelle Osse, Marieke L. de Kam, Jeffrey Damman, Thierry P. P. van den Bosch, Elsa Neubert, Robert Rissmann, Jacobus Burggraaf, Naomi Klarenbeek, Manon A. A. Jansen, Matthijs Moerland

**Affiliations:** 1 Centre for Human Drug Research, Leiden, Netherlands; 2 Leiden University Medical Centre, Leiden, Netherlands; 3 Department of Dermatology, Erasmus Medical Centre, Rotterdam, Netherlands; 4 Department of Pathology and Clinical Bioinformatics, Erasmus Medical Centre, Rotterdam, Netherlands; 5 Leiden Academic Centre for Drug Research, Leiden University, Leiden, Netherlands

**Keywords:** corticosteroids, dermal, human, inflammasome (NLRP3), LPS (lipopolysaccharide), neutrophil extra cellular traps

## Abstract

**Background:**

Intradermal lipopolysaccharide (LPS) administration is a well-established model to investigate Toll-like receptor 4 (TLR4)-mediated inflammation in human skin. Previous studies have shown strong immune activation following LPS injection, including neutrophil influx and cytokine release. However, these studies lacked early time-point assessments, limiting understanding of initial inflammatory kinetics. Additionally, corticosteroids, while known to suppress some inflammatory effects, did not inhibit early neutrophil recruitment.

**Objectives:**

This study aimed to characterize the early innate immune response to intradermal LPS in healthy volunteers, with specific focus on IL-1β, neutrophil infiltration, neutrophil extracellular trap (NET) formation, and inflammasome activation. We also evaluated the impact of oral prednisolone on these early responses.

**Methods:**

In a randomized, placebo-controlled, double-blind trial, 36 healthy male volunteers received oral prednisolone or placebo (1:1) for 2.5 days prior to LPS challenge. Participants were injected with intradermal LPS (5 ng/injection) on the volar forearm and monitored at multiple time points (1, 3, 6, 24 h). Vascular responses were assessed by multispectral imaging and laser speckle contrast imaging. Immune responses were measured through cytokine analysis of suction blister fluid, flow cytometry, and immunohistochemistry of skin punch biopsies.

**Results:**

Intradermal LPS triggered a rapid increase in skin perfusion and erythema, detectable from 1 hour post-injection. IL-8 peaked at 1 hour, while IL-6 and IL-1β peaked at 3–6 hours. A marked neutrophil influx and NET formation were observed as early as 1 hour, with peak NET formation at 24 hours. Classical monocyte infiltration followed neutrophil recruitment. NLRP3 inflammasome activation, as evidenced by ASC and NLRP3 expression, peaked at 3 hours. Prednisolone reduced vascular responses and infiltration of intermediate monocytes but had limited effect on early cytokine levels, neutrophil influx, NET formation, or inflammasome activation.

**Conclusions:**

The intradermal LPS model reveals a rapid sequence of inflammatory events, beginning within one-hour post-challenge. While prednisolone attenuates some late-phase responses, early neutrophil-driven and inflammasome-mediated events remain largely unaffected. These findings enhance the utility of this model for studying early innate immune responses and evaluating new anti-inflammatory therapies targeting IL-1β and NET formation specifically.

## Introduction

Inflammation is a physiological response to pathogens or tissue injury, aimed at restoring homeostasis through elimination of the harmful agents and the initiation of tissue repair processes (1). An excessive or poorly regulated immune response can be harmful to the host as can be observed in a wide range of inflammatory or autoimmune disorders (2). Inflammation can be broadly divided into distinct phases, including the acute phase, resolution, and tissue remodeling; disruption in the transition between these phases may result in chronic inflammation. In the present study, we focus exclusively on the acute phase. To investigate the physiology of inflammation, pro-inflammatory stimuli are commonly employed. Intradermal lipopolysaccharide (LPS) administration has proven to be a valuable tool to study the peripheral toll-like receptor (TLR) 4-mediated inflammatory response in healthy volunteers (3, 4). The cellular response starts with a rapid influx of neutrophils followed by different monocyte subsets, dendritic cells and T cells. The evoked dermal skin reaction shows activator protein (AP)-1, nuclear factor-κB (NFκB), and interferon regulatory factor (IRF)-driven cytokines such as interleukin (IL)-1β, IL-6, IL-8, IFN-γ and tumor necrosis factor (TNF). Corticosteroids, both topical clobetasol and oral prednisolone, have been shown to reduce the LPS-induced clinical inflammatory response i.e. erythema, temperature, perfusion and the cellular response including monocyte subsets, dendritic cells and natural killer (NK) cells. Interestingly, corticosteroid treatment did not substantially suppress the TLR4-mediated acute neutrophil influx after intradermal LPS injection but did reduce the number of neutrophils after 24 hours (4).

The earliest timepoint at which LPS-driven skin responses were evaluated in earlier studies was 3 hours after LPS administration (3). At that time point, clinical responses (erythema, skin perfusion) and immune responses (neutrophil attraction) were already peaking, precluding assessment of the response onset or true peak timing and level. Therefore, we did a follow-up study with a focus on the initial hours after intradermal LPS injection. Specific attention was paid to innate immune responses occurring early in the TLR4-mediated response: IL-1β release, neutrophil attraction and the formation of neutrophil extracellular traps (NETs), events that drive the subsequent phases of the inflammatory response, and critical targets for future immunomodulatory drugs for autoimmune diseases. The NLRP3 inflammasome, alongside the NLRP1 inflammasome, plays a key role in the activation of IL-1β in skin, a crucial mediator in inflammatory responses. The NLRP3 inflammasome has been extensively studied in neurodegenerative disorders like Alzheimer’s and Parkinson’s disease (5), but the NLRP3 alongside the NLRP1 inflammasome is also important, in autoimmune skin diseases such as psoriasis and vitiligo (6). Innate immune cells such as monocytes but also keratinocytes express NLRP3 and thus a human skin challenge model could serve as possible new model to investigate NLRP3-mediated disorders and anti-NLRP3 based therapies (7, 8). Research on NETs is rapidly advancing and drugs designed to selectively target NET formation without impairing other neutrophil functions may have therapeutic value in managing autoimmune and auto-inflammatory disorders, including rheumatoid arthritis, systemic lupus erythematosus and hidradenitis suppurativa (9, 10). This research would benefit largely from the availability of human *in vivo* tissue-based models for NET evaluation, which highlights the potential of the intradermal LPS model. In this study, oral prednisolone was included as anti-inflammatory treatment to assess the effect of corticosteroids on early components of this innate immune response. Our overall aim was to further characterize the early LPS-driven skin response, thereby optimizing and expanding the intradermal LPS challenge as human *in vivo* model for TLR4-mediated tissue inflammation, and to investigate the impact of corticosteroid treatment on these processes.

## Materials and methods

This study was conducted from March 2021 to September 2021 at the Centre for Human Drug Research (CHDR), The Netherlands, according to the Dutch Act on Medical Research involving Human Subjects (WMO). The study protocol was registered in the EudraCT database (number 2019-003567-21). The study protocol was approved by a Medical Ethics Committee (Stichting Beoordeling Ethiek Biomedisch Onderzoek, Assen, The Netherlands) prior to the start of the clinical phase. Participants gave written informed consent before any study-related procedures were undertaken.

### Study design and subjects

**Figure 1:**
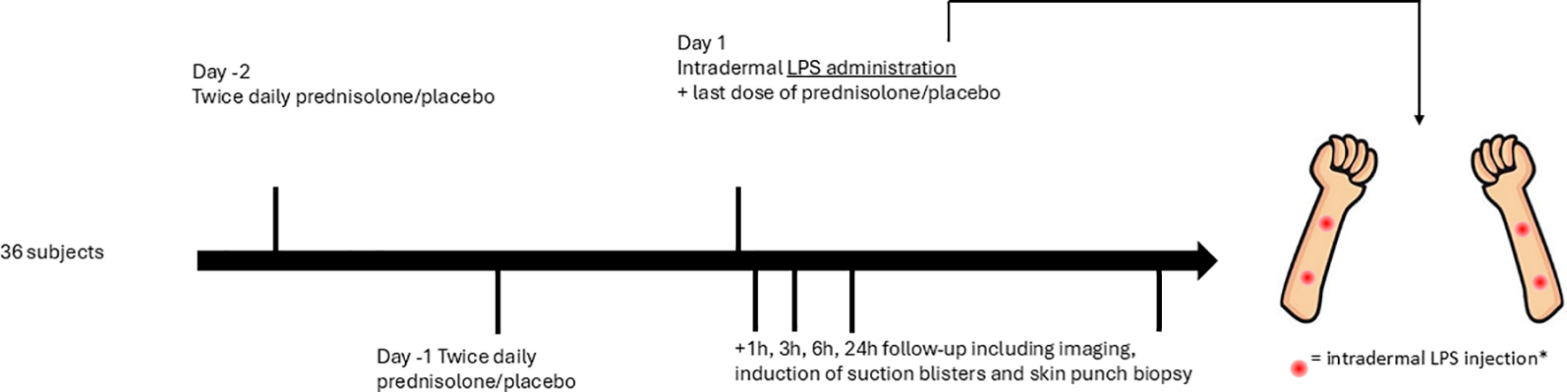
Graphical display of the study design. Subjects were pre-treated with oral prednisolone at 0.25 mg/kg body weight or placebo for two-and-a-half days prior to LPS administration. Follow-up took place 1h, 3h, 6h, and 24h after LPS administration. *Subjects 1–12 received two intradermal LPS injections and subjects 13–36 received four intradermal LPS injections.

This was a randomized, placebo-controlled, double-blind trial in which 36 non-smoking healthy males (Fitzpatrick skin type I-III), age 18–45 years, were included. Participants with any immune disease or recent infection were excluded. All subjects received two-and-a-half day twice daily pre-treatment with oral prednisolone dosed at 0.25mg/kg body weight per administration prior to LPS administration. Subjects were randomized 1:1 to prednisolone or placebo. On the day of LPS administration subjects received the final administration of prednisolone. Participants received a maximum of four intradermal LPS injections (5 ng LPS in 50 µL 0.9% NaCl solution per injection) in the volar forearm. Five timepoints (including baseline *i.e.* pre-LPS) were assessed in total and participants contributed with a maximum of 4 invasive assessments (i.e. skin punch biopsies and suction blisters). See **Figure 1** for a graphical display of the study design. LPS injections took place in the morning whenever possible to minimize the possible effect of the circadian rhythm.

### Dermal imaging

Erythema and perfusion of the skin was evaluated before dosing and at either 1, 3, 6, or 24 hours after LPS administration (**Supplementary Table S1**). Erythema was assessed by multispectral photo analysis (Antera 3D, Miravex, Ireland) and perfusion by laser speckle contrast imaging (LSCI; PeriCam PSI System, Perimed Jäfälla, Sweden). Skin assessments were performed in a climate-controlled room with temperature between 19 and 21 degrees Celsius after 20 minutes of acclimatization.

### Suction blister induction and analysis

At the indicated timepoints (**Supplementary Table S1**) a suction blister was raised over the marked injection site or untreated (baseline) area. The induction of suction blisters was performed according to the method published by Motwani et al. (11) The blister fluid was collected immediately after the blister was formed in a V-bottom plate containing 50 μL 3% sodium citrate (Sigma) in PBS (Gibco) and kept on ice. The plate was centrifuged, and supernatant was weighed to estimate the volume and then frozen at -80 °C for cytokine analysis (Meso Scale Discovery, Rockville, Maryland, USA), the following cytokines and molecules were analyzed: IL-1β, IL-6, IL-8, IFN-γ, ASC, and NLRP3. The pellet was resuspended in RoboSep buffer (Stemcell). A cocktail of fluorescent antibodies for cell surface markers was added to the cells and incubated for 30 minutes on ice. Stained samples were washed with PBS and measured with a MACSQuant 10 (Miltenyi Biotec GmbH). Flow cytometry data were analyzed with Flowlogic 7.1 (Inivai). Concomitant with blister fluid collection, peripheral blood was drawn by venepuncture using sodium heparin containing tubes (Vacutainer BD). 100 µl whole blood was treated with red blood cell lysis buffer (eBioscience) and washed with PBS and resuspended in RoboSep buffer. Staining was similar to previously mentioned blister cells. The following antibodies were used for both blister fluid and whole blood: CD14 FITC (clone REA599, cat# 130-119-616, Miltenyi Biotec), HLA-DR APC-Vio770 (clone REA805, cat# 130-111-790, Miltenyi Biotec), CD45 VioGreen (clone REA747, cat# 130-110-638, Miltenyi Biotec), CD66b PE-Vio770 (clone REA306, cat# 130-110-538, Miltenyi Biotec), CD16 BV605 (clone 3G8, cat# 302040, BioLegend), Siglec-8 PE-Vio615 (clone REA1045, cat# 130-117-903, Miltenyi Biotec), and 7AAD (cat# 130-111-568, Miltenyi Biotec). Cell populations (single live cells) were classified based on the following profile: CD45^+^Siglec8^-^CD16^+^CD66b^+^ neutrophils, CD45^+^HLA-DR^+^CD14^+^CD16^-^ classical monocytes, CD45^+^HLA-DR^+^CD14^+^CD16^+^ intermediate monocytes, CD45^+^HLA-DR^+^CD14^-^CD16^+^ non-classical monocytes. An overview of the gating strategy can be found in **Supplementary Figure 1**.

### Skin punch biopsy collection and analysis

Skin punch biopsies (3 mm) were collected under local anesthesia (lidocaine/adrenaline, Lignospan, Septodont) and snap frozen immediately using liquid nitrogen as previously described (12). The biopsies were stored at −80 °C until analysis. Immunohistochemistry was performed for the following targets: ASC (polyclonal AL177; AdipoGen), IL-β (polyclonal ab9722; Abcam), IL-18 (polyclonal HPA003980; Atlas Antibodies), NLRP3 (polyclonal HPA012878; Atlas Antibodies), Elastase (polyclonal HPA066836; Atlas Antibodies), MPO (polyclonal 760–2659; Cell Marque Sigma-Aldrich), and H3Cit (polyclonal ab5103, Abcam). The slides were scored qualitatively on a 6-point scale (negative, minimal, few, moderate, many or excessive) blinded. Immunofluorescence (IF) double staining was performed for CD14 (clone EPR3653; Cell Marque Sigma-Aldrich) and CD16 (monoclonal DJ130c; Santa Cruz Biotechnology). All IF slides were scanned at 20x magnification using an Axio lmager 2 Fluorescence color microscope (ZEISS, Oberkochen, Germany). The biopsy samples were analyzed using QuPath Quantitative Pathology & Bioimage Analysis software (Version 0.3.0), an open software platform for bioimage analysis.

### Statistics

Repeated measured pharmacodynamic continuous data were analyzed with a mixed effects model with fixed factors treatment, time and treatment by time and random factor subject and the, if available, average value pre-treatment as covariate. The following contrast was calculated within the model: prednisolone vs. placebo. Statistical inference for the contrast was based on a Wald t-test with Kenward–Roger approximated degrees of freedom. All calculations were performed using SAS for windows V9.4 (SAS Institute, Inc., Cary, NC).

## Results

### LPS drives an acute vascular response

**Figure 2:**
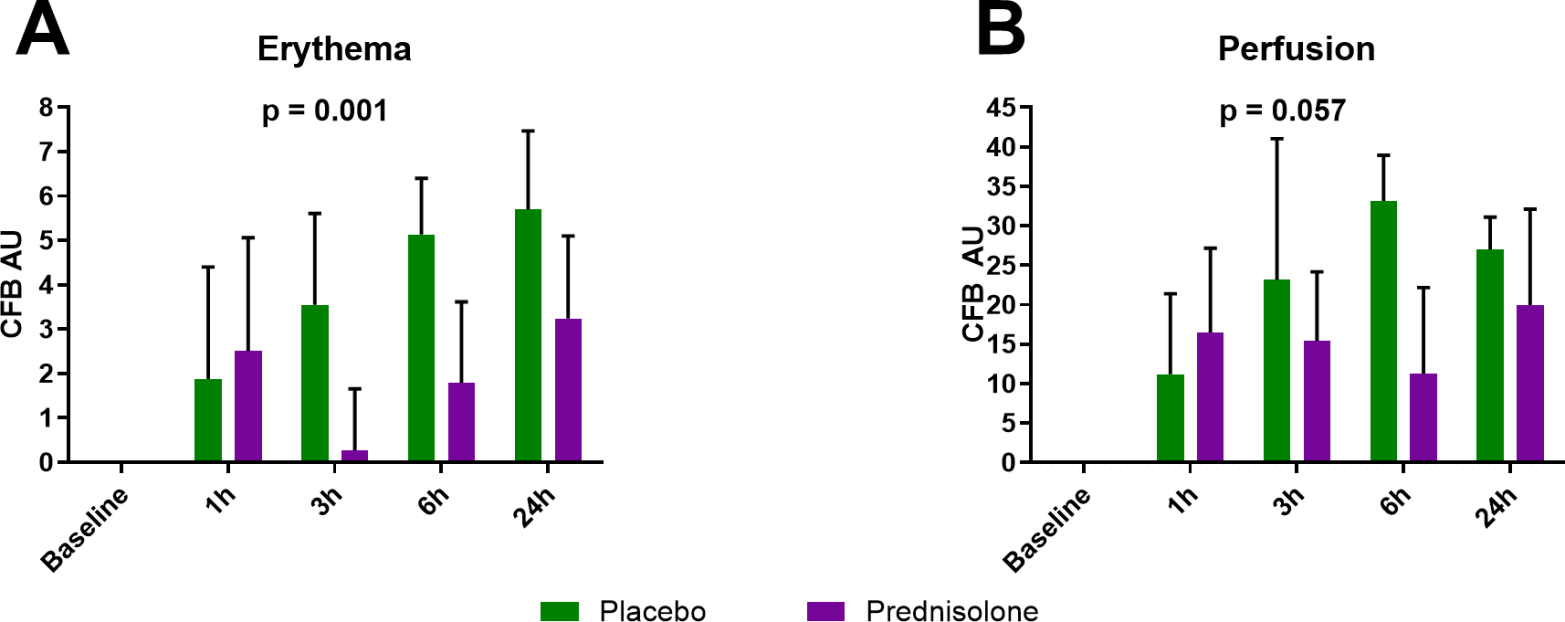
Prednisolone successfully reduced the clinically observed inflammatory response. The clinical inflammatory response was analyzed by quantifying erythema **(A)** and perfusion **(B)**. All data are presented as mean ± SD change from baseline. Erythema p = 0.001 (treatment effect over the observation period), Perfusion p = 0.057 (treatment effect over the observation period).

The vascular response to intradermal LPS was quantified as erythema and perfusion. At one hour after LPS administration, the earliest measured time point, a marked increase in erythema and perfusion was observed (**Figures 2A, B**). For erythema the maximum response was noted at 24 hours after LPS administration (**Figure 2A**), whereas perfusion peaked at 6 hours after LPS administration and diminished slightly at the 24 hour time-point (**Figure 2B**). In alignment with our previous study, prednisolone successfully reduced the clinical response to intradermal LPS administration (**Figures 2A, B**). Interestingly, at one hour after LPS administration no substantial difference between prednisolone and placebo could be observed, but from three hours after LPS administration and onward, prednisolone reduced the LPS-driven increase in erythema (p = 0.001) without significantly impacting LPS-driven perfusion (p = 0.057) when evaluated over the observation period.

### The inflammatory cytokine response to intradermal LPS is marked by early IL-6 and IL-8 production and is not decreased by prednisolone

**Figure 3:**
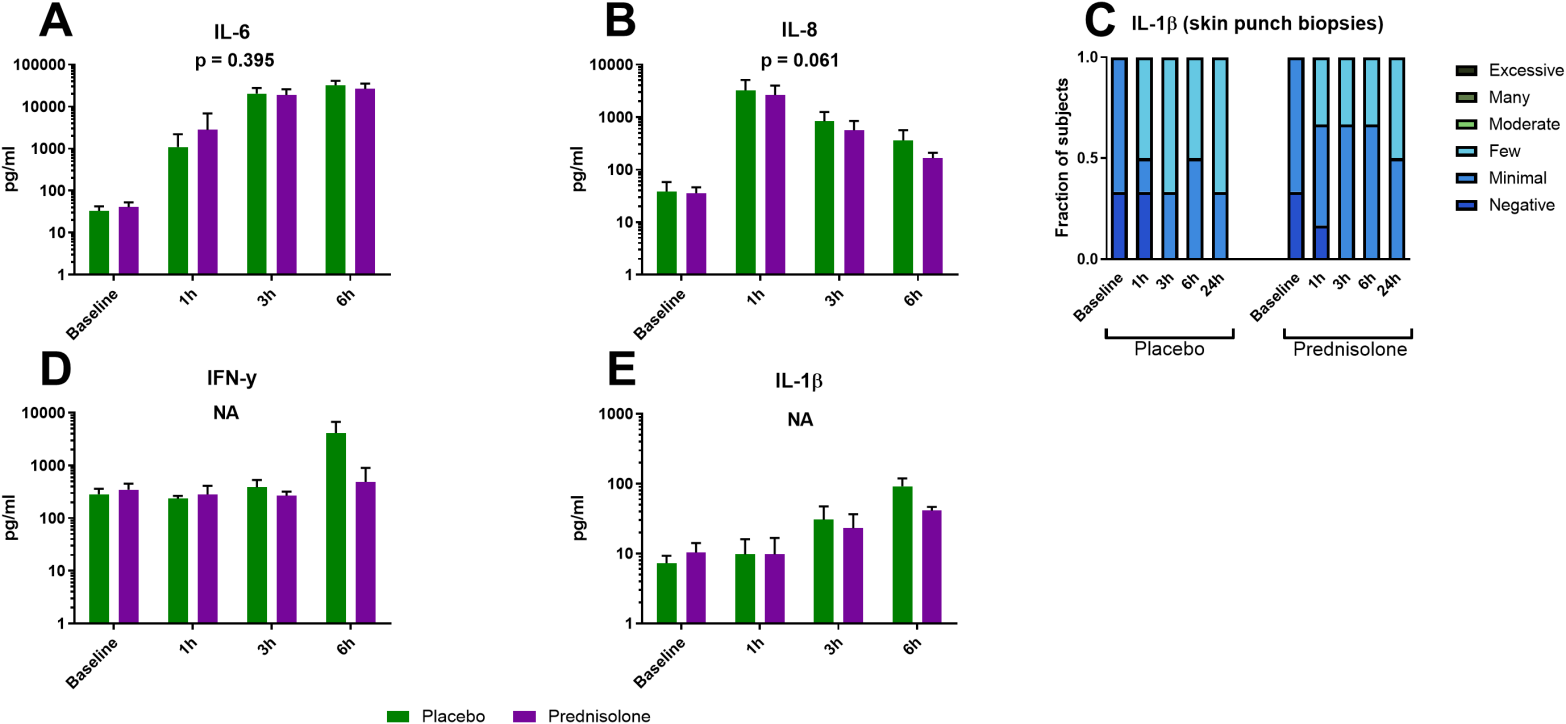
Intradermal LPS induces a strong cytokine response and prednisolone does not affect this response. Cytokines were measured in blister fluid by MSD **(A, B, D, E)**, presented as mean ± SD) and in skin punch biopsies by immunohistochemistry **(C)**. NA, no statistical analysis applied.

Intradermal LPS induced a strong response of IL-6 (**Figure 3A**) and IL-8 (**Figure 3B**) in blister fluid. The IL-8 response peaked immediately at our earliest time point namely 1 hour after LPS administration (**Figure 3B**). IL-6 was also already markedly increased at 1 hour but peaked 3–6 hours after LPS administration (**Figure 3A**). IL-1β levels were highest at 6 hours after LPS administration (**Figure 3E**) and stayed present also at the 24 hour time point as measured by immunohistochemistry in skin punch biopsies (**Figure 3C**). IFN-γ levels were only slightly elevated from baseline levels at 6 hours after LPS administration (**Figure 3D**). IL-18 levels were measured but below the LLOQ (200 pg/mL) which was confirmed by immunohistochemistry staining in skin punch biopsies (no positive staining, data not shown). When analyzed over the full time profile prednisolone did not reduce the inflammatory cytokine response (IL-6 and IL-8), although data suggest moderate cytokine response suppression at the 6 hour timepoint (**Figures 3A, B**). No statistical analysis was performed for IFN-γ and IL-1β because a high number of samples were below the LLOQ.

### LPS induces a rapid neutrophil response followed by appearance of classical monocytes and is not modulated by prednisolone

**Figure 4:**
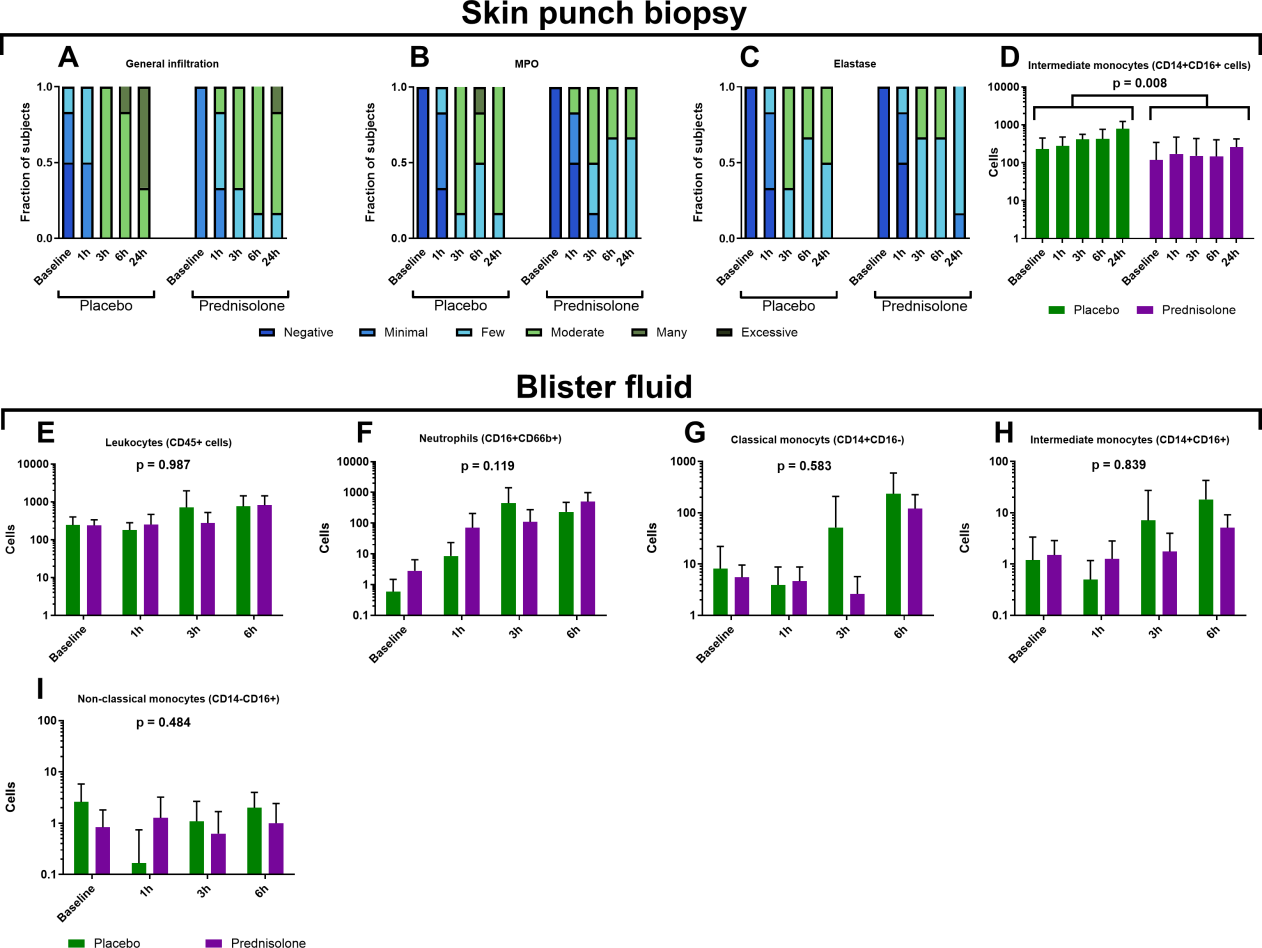
Intradermal LPS administration leads to a general inflammatory cell influx **(A, E)** including monocyte subsets **(D, G–I)** and neutrophils **(B, C, F)**. Panels **(A–C)** were measured by immunohistochemistry and **(D)** by immunofluorescence in skin punch biopsies. Panels **(E–I)** were measured by flow cytometry in blister fluid. Prednisolone only significantly reduced the intermediate monocyte response measured by immunofluorescence in skin punch biopsies [**(D)**, p = 0.008, treatment effect over the observation period]. Data is presented as fraction of subjects **(A–C)** and mean + SD **(D–I)**.

In line with our previous work, intradermal LPS injection led to an increase in circulating leukocytes which mainly consisted of neutrophils (**Supplementary Figure 2**) (3). LPS induced a local general inflammatory cell infiltration that was maximum 24 hours after LPS administration (**Figure 4A**). A rapid influx of CD16^+^CD66b^+^ neutrophils in blister fluid was observed (**Figure 4F**) which was already noticeable one hour after LPS administration and more pronounced after 3 hours. The neutrophil influx as quantified in blister fluid mirrored the presence of MPO and elastase positive cells in skin punch biopsies (**Figures 4B, C**). From our previous studies we know that monocyte influx was maximum at 24h and 48h hours after LPS administration and in line with those findings we only observed a slight increase in CD14^+^CD16^-^ classical monocytes starting at 3 hours after LPS administration in blister fluid (**Figure 4G**) and a peak of CD14^+^CD16^+^ intermediate monocytes was visible at 24 hours after LPS administration as quantified by immunohistochemistry (**Figure 4D**) (3, 4).

Prednisolone treatment did not substantially alter the early cellular response following intradermal LPS administration (**Figures 4A, E**). By visual inspection of the data, prednisolone appeared to slightly reduce the general inflammatory cell infiltration (**Figure 4A**) and prednisolone significantly reduced the amount CD14^+^CD16^+^ intermediate monocytes (p = 0.008, **Figure 4D**). Visual inspection of the data hints towards a slight reduction in MPO and elastase positive cells at 6h and 24h after LPS administration in the prednisolone treated subjects compared to placebo treated subjects (**Figures 4B, C**). No statistically significant changed were seen in the early cellular response between prednisolone treated subjects and placebo treated subjects (**Figures 4E–I**), the analysis was performed over the whole time course.

### Intradermal LPS administration triggers the formation of neutrophil extracellular traps

**Figure 5:**
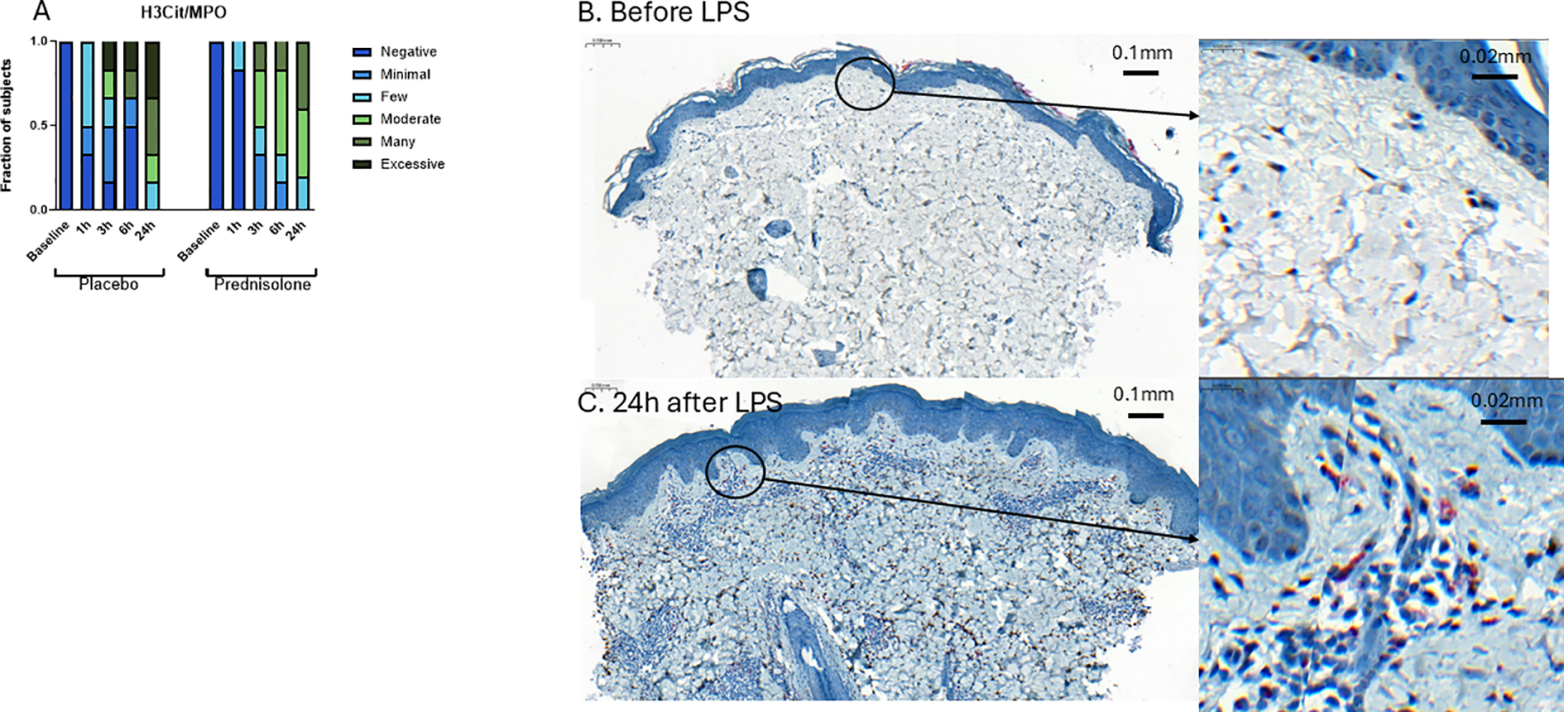
Formation of NETs after intradermal LPS administration. NETs were identified by co-staining of H3Cit/MPO by immunohistochemistry where MPO shows a brownish-black color and H3Cit a pinkish color. Panel **(A)** shows the amount of H3Cit/MPO positive cell in placebo and prednisolone treated subjects. Panels **(B, C)** show a representative slide of skin punch biopsies stained for H3Cit/MPO before and 24 hours after LPS administration.

To better understand the kinetics of the local neutrophil response induced by intradermal LPS, and to investigate whether intradermal LPS administration would trigger the formation of neutrophil extracellular traps (NETs), we quantified neutrophils in blister fluid and stained skin punch biopsies for MPO, elastase and H3cit/MPO. In line with our previous work, LPS administration leads to an influx of neutrophils that were already visible as early as one hour after injection, as measured by flow cytometry in blister fluid (**Figure 4F**). This influx of neutrophils was also visible in the skin punch biopsies where MPO and elastase were used as neutrophil markers (**Figures 4B, C**). The presence of NETs was investigated by staining for H3cit/MPO3 and samples were scored positive if H3cit was found together with or in close proximity to MPO. At baseline no NETs were detected (**Figures 5A, B**). Already at 1h after LPS administration NETs were present, becoming increasingly more abundant throughout the time course (**Figure 5A**). The formation of NETs was most abundant 24h after LPS administration (**Figures 5A, C**). Prednisolone did not substantially reduce this NET response to LPS (**Figure 5A**).

### Intradermal LPS administration induces an IL-1β response and upregulation of NLRP3

**Figure 6:**
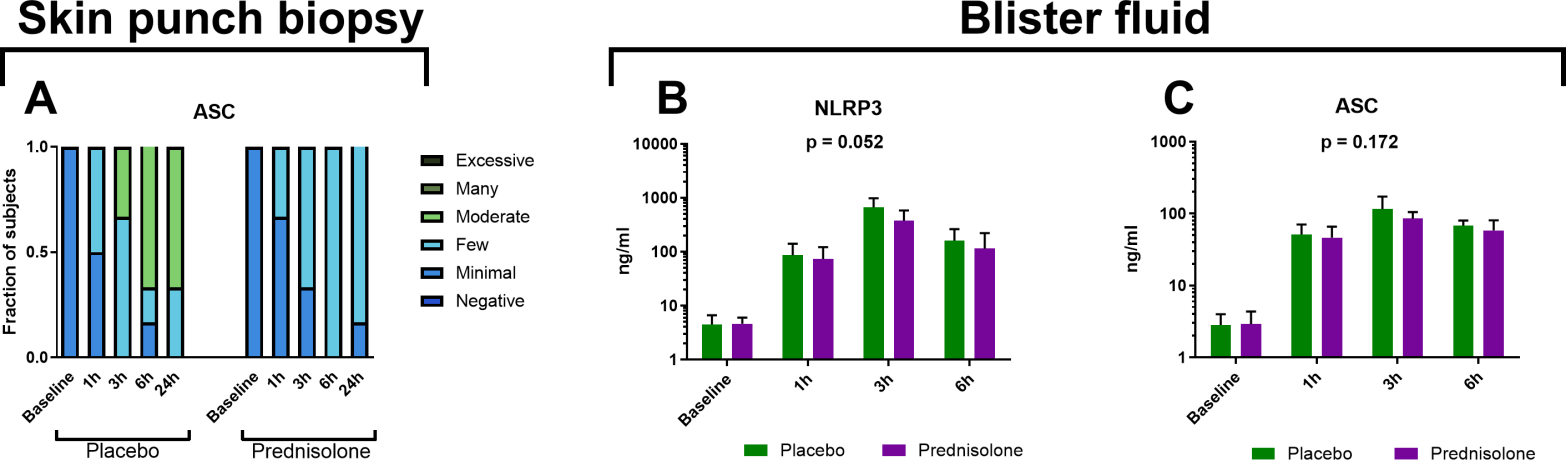
LPS induces the expression of NLRP3 **(B)** as measured in blister fluid by MSD) and subunit ASC **(A)** as measured in skin punch biopsies by immunohistochemistry and figure 6C measured in blister fluid by MSD). Prednisolone did not significantly influence the NLRP3 [**(B)**, p = 0.052 over the observation period] or ASC expression [**(C)**, p = 0.172 over the observation period].

To investigate whether the previously observed IL-1β response to LPS was mediated by the NLRP3 inflammasome we quantified NLRP3 inflammasome subunits ASC and NLRP3 in both blister fluid and skin punch biopsies. We failed to positively stain NLRP3 in skin punch biopsies (data not shown), most likely due to technical issues because we did quantify NLRP3 protein in the blister fluid (**Figure 6B**). NLRP3 was absent at baseline and peaked relatively early at 3 hours after LPS administration. Prednisolone slightly dampened the observed NLRP3 response, although not statistically significant (p = 0.052). ASC, a subunit of the NLRP3 inflammasome, was detectable in both skin punch biopsies (**Figure 6A**) and blister fluid (**Figure 6C**). Like NLRP3, ASC peaked at 3 hours after LPS administration (**Figure 6B**) and prednisolone seemed to damp the ASC response slightly (**Figures 6C, D**), although not statistically significant (p = 0.172).

### Safety

Adverse events were registered and coded according to the Medical Dictionary for Regulatory Activities (MedRA). In total five adverse events occurred in the placebo treated subjects (headache in three subjects, fatigue in one subject, abdominal pain in one subject). A total of 11 adverse events occurred in prednisolone treated subjects (fatigue in two subjects of which one experienced fatigue on two separate occasions, abdominal pain in two subjects, nausea in one subjects on two separate occasions, headache in two subjects of which one experienced headache on two separate occasions, dry throat in one patient. All adverse events were classified as mild and one subject used paracetamol for headache and one subject used ibuprofen for headache in the evening after he completed all study related procedures.

## Discussion

The aim of this study was to further characterize the acute inflammatory response to intradermal LPS administration in healthy volunteers with specific attention to the early inflammatory response. Furthermore, the effect of prednisolone treatment on this early TLR4-mediated response was investigated. The vascular response (erythema and perfusion) was already visible from 1 hour after LPS administration. At that time point, no noticeable difference between placebo and prednisolone treated subjects was observed (**Figure 2A**). From 3 hours onwards a clear reduction in erythema and perfusion was observed in the prednisolone treated subjects, in line with the prednisolone effects on dermal LPS responses reported earlier (4). Interestingly, in the prednisolone treated subjects erythema was less at 3 hours than at 1 and 6 hours, indicating potentially different mediators for the early (<6 hours) and later (>6 hours) vascular responses. Presumably, the rapid erythema and perfusion present at 1 hour after LPS administration is mediated through early non-inflammatory vasoactive mediators that are not susceptible to the effects of prednisolone.

In our previous study IL-6 and IL-8 kinetics seemed to follow a similar pattern but here we showed that intradermal LPS administration induced a rapid IL-8 response that already peaks 1 hour after administration and declines rapidly thereafter (**Figure 3B**). This is in contrast with the IL-6 kinetics which is present at 1 hour but rapidly increases at 3 hours and peaks at 6 hours (**Figure 3A**). Prednisolone treated subjects did not show a statistically significant reduction in IL-6 or IL-8 response (**Figures 3A, B**), in line with earlier observations where IL-6, IL-8 but also TNF (not included in this study) were not inhibited by prednisolone (4). Potential candidates for the IL-6 and IL-8 production include keratinocytes, fibroblasts and endothelial cells along with neutrophils that were increasingly present from 1 hour after LPS administration (**Figure 4F**). Keratinocytes and fibroblasts, the most abundant cell types in the epidermis and dermis respectively, are highly immunocompetent and can produce various inflammatory cytokines including IL-6 and IL-8 (13, 14). In contrast to our *in vivo* data, others showed that prednisolone was able to inhibit IL-6 production by UV-B exposed keratinocytes *in vitro* (15). Neutrophils also produce IL-6 and IL-8 when stimulated by LPS (16) and a reduction in pro-inflammatory IL-1β, IL-8 and TNF was seen in LPS stimulated neutrophils in combination with prednisolone *in vitro* (17). Also in LPS stimulated human skin organ cultures dexamethasone was able to reduce the IL-6 and IL-8 (18). Endothelial cells are also capable of producing IL-6 upon LPS stimulation and corticosteroids have shown to inhibit this repsonse (19). We are unable to explain why in our intradermal LPS model prednisolone (and in a previous study topical clobetasol) is unable to inhibit IL-6 and IL-8 production *in vivo (*4), but the observation underlines the relevance of studying the effects of drugs on leukocyte function *in vivo*, in a peripheral tissue.

In line with our previous results (3, 4) we confirmed that LPS induced an IL-1β response but this time we also quantified the upregulation of two quintessential components of the NLRP3 inflammasome namely NLRP3 and ASC (5). Upregulation of ASC was quantified in both skin punch biopsies and blister fluid (**Figures 6A, C**). Whereas NLRP3 protein was quantified in blister fluid (**Figure 6B**), we failed to quantify NLRP3 in skin punch biopsies (data not shown), most likely due to a technical issue with the staining procedure. In addition to IL-1β we also analyzed the NLPR3 dependent pro-inflammatory cytokine IL-18 (data not shown). In blister fluid all measures were below the LLOQ and in skin punch biopsies all IL-18 stainings were negative. Although IL-18 is constitutively expressed by basal and suprabasal keratinocytes and secreted upon LPS exposure, it can be difficult to measure since it is rapidly neutralized by its natural inhibitor IL-18 binding protein (IL-18BP) (20–22). In humans there is both *in vitro* and *in vivo* evidence that LPS induces a NLRP3 dependent IL-18 response (23–25). Although inflammasome independent IL-1β/IL-18 activation is also possible through active caspase-8 (26, 27) and neutrophil serine proteases (28), these pathways always lead to active IL-1β together with active IL-18. In microglia stimulated with *Staphylococcus aureus*, cathepsin B caused inflammasome independent activation of IL-1β but not IL-18, but upon stimulation active IL-18 was still formed independent of NLRP3 or ASC (29). Thus IL-1β and IL-18 can be activated through different and separate pathways and future studies should confirm our negative IL-18 findings. Our findings of marked upregulation of NLRP3 and ASC combined with IL-1β production suggest that the LPS induced IL-1β response is at least partly mediated through the NLRP3 inflammasome, although future studies should confirm actual activation of the NLRP3 inflammasome. In the current study we only focused the NLRP3 inflammasome but future studies should also include the NLRP1 inflammasome which is particularly important in the pathogenesis of several skin diseases (6, 7).

IL-8, also known as CXCL8, is an important neutrophile attractant, peaking at 1 hour after LPS administration in our study. We observed an acute increase in neutrophils in blister fluid (**Figure 4F**) and MPO/elastase as neutrophil marker in skin punch biopsies (**Figures 4B, C**). LPS not only attracted neutrophils but also triggered the formation of neutrophil extracellular traps (NETs) (**Figures 5A, C**). NETs were already present one hour after LPS administration but became more abundant throughout the observation period and peaked 24 hours after LPS administration. In line with our previous study, prednisolone did not significantly affect the influx of neutrophils, which is not surprising given the fact that prednisolone treatment did not alter the LPS-driven IL-8 response. Prednisolone does not appear to substantially influence NET formation (**Figure 5A**), although data suggest fewer NETs in prednisolone-treated volunteers. Further quantitative research is necessary to validate these results. The role of corticosteroids on NET formation so far has been ambiguous: inhaled corticosteroids were able to reduce plasma NET levels in patients with asthma and chronic obstructive pulmonary disease (COPD) (30), but corticosteroids also induced NET formation in neutrophils from healthy donors (31), these discrepancies might be explained by different doses, treatment duration and/or administration route. The formation of NETs is thought to play an important role in many (autoimmune) disorders including systemic lupus erythematosus and rheumatoid arthritis (9). Studying the formation of NETs in humans can contribute to disease understanding and can lead to new therapeutic targets. We argue that the intradermal LPS challenge as presented in this study can contribute to these aims.

In conclusion, this study provides further insight into the early inflammatory kinetics of intradermal LPS administration. Furthermore, intradermal LPS administration leads to IL-1β production and the formation of neutrophil extracellular traps (NETs). These insights highlight the human intradermal LPS model as a valuable tool for the in-depth investigation of *in vivo* inflammatory pathways in both health and disease. Moreover, it offers a relevant framework to study the regulation and balance of the immune response, which are essential for maintaining homeostasis but may contribute to disease when dysregulated or excessive. Importantly, whereas oral prednisolone treatment suppressed the early vascular response 3 hours after LPS and onwards, it did not substantially modulate early cellular and cytokine responses. This reveals the potential for novel anti-inflammatory compounds to specifically target these early stages of inflammation.

## Data Availability

The raw data supporting the conclusions of this article will be made available by the authors, without undue reservation.

## Supplementary material

Supplementary Figure 1 Gating strategy for the flow cytometry analysis.

Supplementary Figure 2 Circulating leukocytes and neutrophils before prednisolone or placebo, after two-day pretreatment with prednisolone or placebo, and after LPS injections (1h, 3h, 6h, 24h). Data is depicted as box plots. Black dot = mean and white dot = outlier.

Supplementary Table 1 The four different sites per subject were allocated to either a suction blister or skin punch biopsy according to this timetable. *: At these sites non-invasive measures were performed before LPS administration and at the indicating time point.
