# Supplementary figures and images for: Early immune responses to intradermal lipopolysaccharide in healthy volunteers: prednisolone’s impact on TLR4-mediated inflammation
Source: Front Immunol. 2026 May 20;17:1724791. doi: 10.3389/fimmu.2026.1724791 (PMC13229733; doi:10.3389/fimmu.2026.1724791)

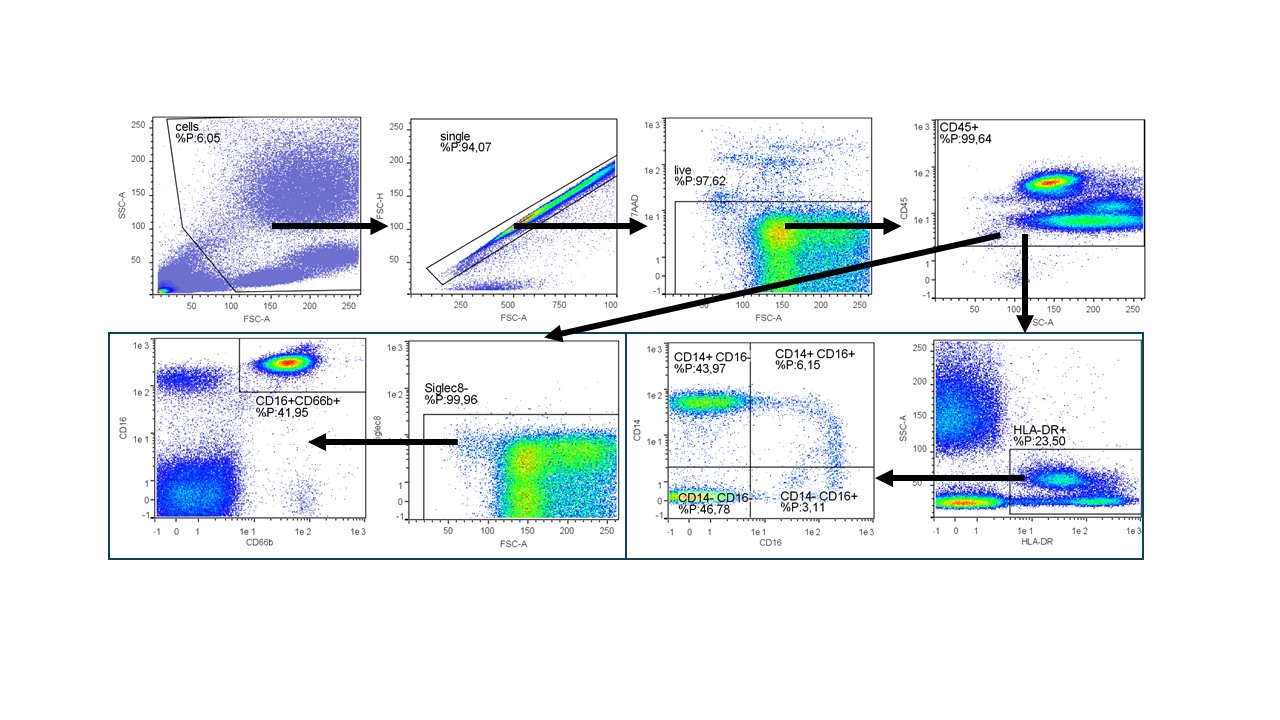

Supplement: Supplementary Figure 1 — Gating strategy for the flow cytometry analysis. [file Supplementaryfile1.jpg]

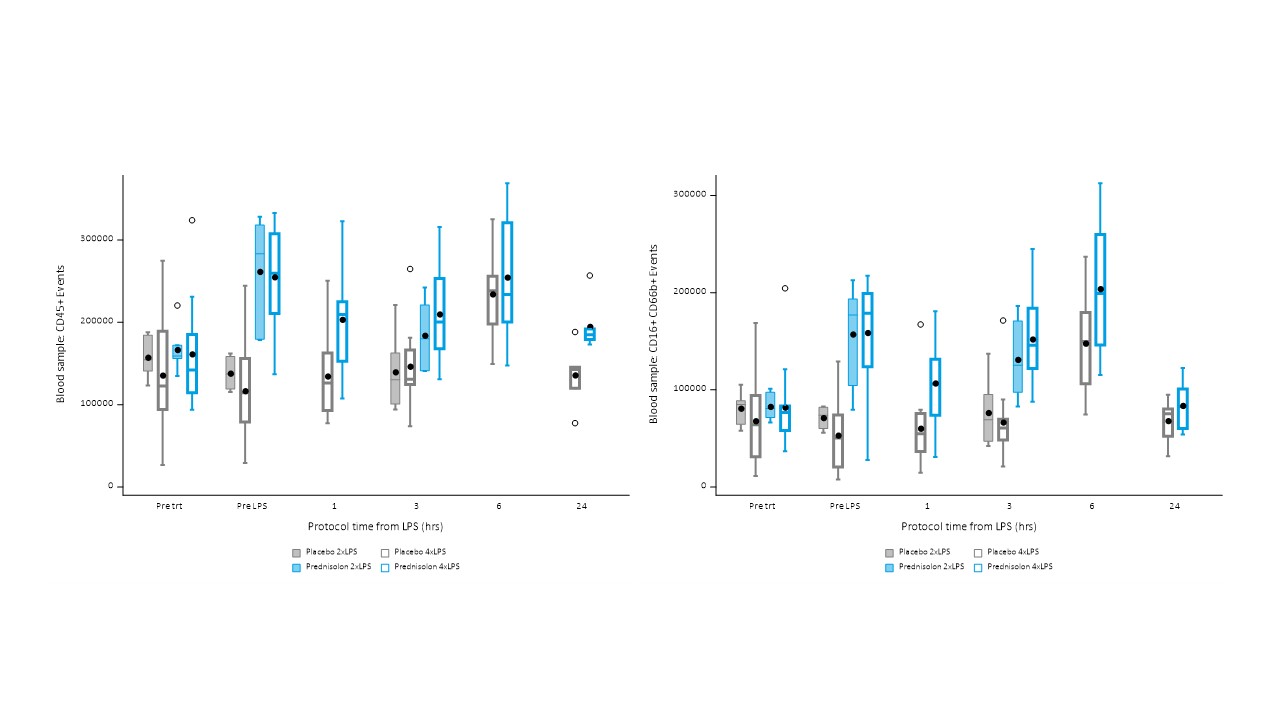

Supplement: Supplementary Figure 2 — Circulating leukocytes and neutrophils before prednisolone or placebo, after two-day pretreatment with prednisolone or placebo, and after LPS injections (1h, 3h, 6h, 24h). Data is depicted as box plots. Black dot = mean and white dot = outlier. [file Supplementaryfile2.jpg]

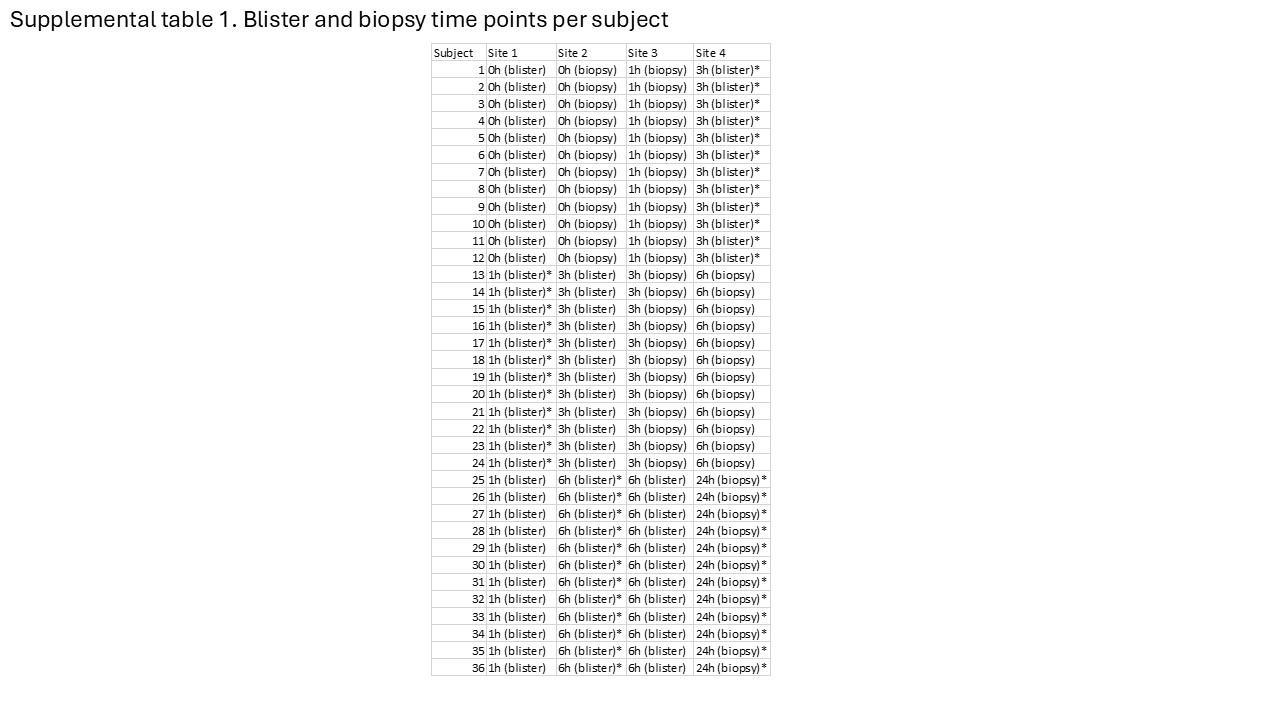

Supplement: Supplementary Table 1 — The four different sites per subject were allocated to either a suction blister or skin punch biopsy according to this timetable. *: At these sites non-invasive measures were performed before LPS administration and at the indicating time point. [file Supplementaryfile3.jpg]
